# Ochratoxin A: Molecular Interactions, Mechanisms of Toxicity and Prevention at the Molecular Level

**DOI:** 10.3390/toxins8040111

**Published:** 2016-04-15

**Authors:** Tamás Kőszegi, Miklós Poór

**Affiliations:** 1 Department of Laboratory Medicine, University of Pécs, H-7624 Pécs, Hungary; 2 János Szentágothai Research Center, Lab-on-a-chip Research Group, H-7624 Pécs, Hungary; 3 Department of Pharmacology and Pharmacotherapy, Toxicology Section, University of Pécs, H-7624 Pécs, Hungary; poor.miklos@pte.hu

**Keywords:** Ochratoxin A, nephropathy, toxicokinetics, cellular effects, albumin binding, flavonoids, prevention

## Abstract

Ochratoxin A (OTA) is a widely-spread mycotoxin all over the world causing major health risks. The focus of the present review is on the molecular and cellular interactions of OTA. In order to get better insight into the mechanism of its toxicity and on the several attempts made for prevention or attenuation of its toxic action, a detailed description is given on chemistry and toxicokinetics of this mycotoxin. The mode of action of OTA is not clearly understood yet, and seems to be very complex. Inhibition of protein synthesis and energy production, induction of oxidative stress, DNA adduct formation, as well as apoptosis/necrosis and cell cycle arrest are possibly involved in its toxic action. Since OTA binds very strongly to human and animal albumin, a major emphasis is done regarding OTA-albumin interaction. Displacement of OTA from albumin by drugs and by natural flavonoids are discussed in detail, hypothesizing their potentially beneficial effect in order to prevent or attenuate the OTA-induced toxic consequences.

## 1. Introduction

Ochratoxin A (OTA) is a well-known and widely-spread mycotoxin all over the world [1]. Ochratoxins (A, B, and C) are secondary metabolites of *Pencillium* and *Aspergillus* micro fungi of which mainly the A form exerts hazardous effects in animals and in humans, as well [1,2,3]. OTA was first found in the Balkan region; however, it can be detected practically in all territories, it is accumulated in animal feed and in human food due to the favorable weather conditions and microclimate, and/or to improper storage of food components [4]. OTA is present at all stages of the food chain (cereals, meat, fruits, wine, beer, coffee, *etc.*) [5,6,7,8,9,10], and based on previous studies its presence may be associated with the chronic tubulo-interstitial kidney disease called Balkan Endemic Nephropathy (BEN) [11,12,13,14]. BEN is a chronic progressive disease with a period of 6–10 years leading to irreversible kidney failure. However, apart from long-term OTA exposition in the endemic areas, some other potential causative factors are postulated in the development of BEN: aristolochic acid, heavy metal intoxication, selenium deficient diet, and genetic predisposition [11,12,15]. Due to its high heat stability, complete removal of OTA from food is practically impossible [16], although several approaches exist for reducing OTA contamination [17,18,19].

After the absorption of OTA from the gastrointestinal tract, it binds primarily to albumin with high affinity, which results in its very long half-life (from a few days to one month, depending on species). In healthy human populations the plasma concentration of OTA ranges from some hundreds of pmol/L to a few nmol/L, while in endemic areas it can exceed 100 nmol/L [20,21,22,23]. Due to the pKa values of OTA (4.2–4.4 and 7.0–7.3, the carboxyl group and the phenolic hydroxyl group, respectively) [24], at physiological pH the toxin is present in monoanionic (OTA^−^) and in dianionic (OTA^2−^) forms [25,26]. In the circulatory system OTA is almost completely bound to albumin. The extent of albumin binding determines the plasma half-life of OTA, being the longest in humans (compared to other species) of approximately one month [27,28]. Albumin binding strongly affects toxicokinetics of OTA: in albumin deficient rats the toxin excretion through the kidneys was 20–70-fold faster than in normal rats [29]. The increased elimination rate might reduce the chronic exposure of the target cells (mainly kidney tubule and liver cells). Since albumin is the key transport protein of the toxin in the circulatory system, and because of the fact that the association constant of the OTA-albumin complex is extremely high (more than 10^7^ L/mol) [25,30,31,32], extensive studies are known characterizing the albumin binding of OTA. Although glomerular filtration of OTA is strongly limited due to its albumin binding, the small filtrated and secreted fraction is partially reabsorbed [1], which might help the accumulation of the toxin in the kidney tubule cells.

OTA toxicity is strongly correlated with the occurrence of BEN [9,33,34]; however, its mechanism of action is very complex [35,36,37,38,39]. It is thought to be carcinogenic, teratogenic, hepatotoxic, neurotoxic, and immunotoxic, based on *in vitro* and on animal studies [1,40]. IARC (International Agency for Research on Cancer) categorizes OTA as a member of 2B subgroup which means that, based on animal studies, OTA is a potential human carcinogen [34,41]. The NCI/NTP (National Cancer Institute/National Toxicological Program) renders OTA to be the most potent renal carcinogen in rodents ever studied [42]. In fact, the incidence of upper urinary tract tumors in endemic regions of Bulgaria is 90-fold higher compared to that of non-endemic regions [43]. In addition to BEN, some studies make emphasis on the role of OTA in the development of Tunisian Nephropathy [44], gastric and esophageal tumors in some regions of China [45,46], as well as testicular cancer [47].

In spite of the several known hypotheses, the mode of action and prevention, or at least attenuation of OTA toxicity, are only partially understood. The present review focuses on the basic knowledge related to Ochratoxin A and on the current information on its molecular and cellular interactions, and also on the attempts to prevent/attenuate its toxicity at the molecular and cellular level.

## 2. Chemistry of Ochratoxins

**Figure 1:**
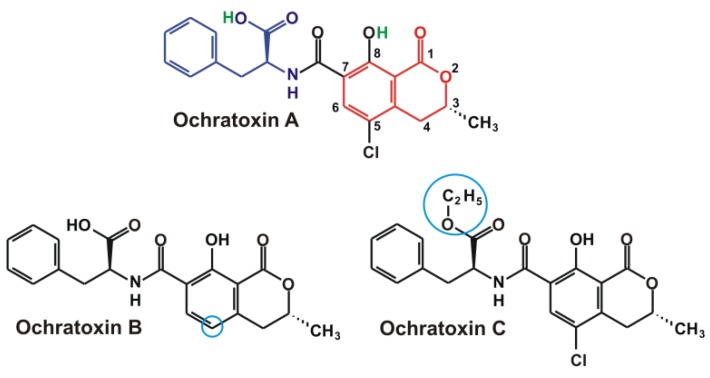
Chemical structures of Ochratoxin A (dark blue: phenylalanine part, red: dihydro-isocoumarin ring, green: acidic hydrogens), B, and C. The highlighted structures are characteristic to the three different ochratoxin molecules (light blue).

Ochratoxins (Ochratoxin A: OTA, Ochratoxin B: OTB and Ochratoxin C: OTC) are toxic metabolites of different fungi; their structure consists of a dihydro-isocoumarin moiety linked with a phenylalanine through an amide bond (Figure 1). Furthermore, OTA and OTC contain a parachlorophenol part as well. OTA (C_20_H_18_ClNO_6_; IUPAC name: *N*-{[(3R)-5-chloro-8-hydroxy-3-methyl-1-oxo-3,4-dihydro-1*H*-isochromen-7-yl]carbonyl}-*L*-phenylalanine; molecular weight: 403.8) is a white, odorless, heat stable, crystalline solid agent (melting point: 168–173 °C) with poor aqueous solubility [3,48]. OTA does not completely disappear during baking [49]; furthermore, OTA resists against three hours of high pressure steam sterilization at 121 °C [50], and even at 250 °C it is only partially degraded [51]. Even during coffee roasting OTA is only partially decomposed [52,53]; one of the identified products is 14-(*R*)-ochratoxin A (about 25%) that shows slightly lower cytotoxicity *in vitro* [54]. Due to the structure of OTA, the mycotoxin exhibits strong fluorescence property [25,26,30]. Depending on the microenvironment, OTA exists in non-ionic, monoanionic (OTA^−^), and dianionic (OTA^2−^) forms.

## 3. Dietary Sources of OTA

Ochratoxin A occurs in wheat, fruits, oilseeds, and animal feed resulting in its presence in milk, meat, and even in eggs [1,3]. Therefore, many drinks (e.g., wine, beer, coffee, tea, milk, *etc.*) [55,56,57], as well as common meals (bakery, meat, and dairy products) [1,40] contain more or less amounts of OTA. Furthermore, recent studies also highlighted its presence in herbal medicines [58,59,60], food coloring agents [61], spices [62,63,64], and even in bottled water [65]. The wide occurrence of OTA and its high thermal stability makes the eradication of OTA from the food chain very difficult.

## 4. Toxicokinetics of OTA

### 4.1. Absorption

The amount of absorbed toxin is species-dependent; in pigs it is about 60%, while in rodents it is much lower [27,66]. The oral bioavailability of OTA is the highest in humans, approximately 93% [27,28]. Based on data of animal experiments, the non-ionic and monoanionic forms of OTA are absorbed from the stomach and the jejunum without known specific transport mechanisms [67,68,69]. On the other hand, the MRP2 multidrug resistance efflux transporter might slow down its absorption from the small intestine due to the transport of OTA back to the intestinal lumen [70]. Furthermore, OTA is also a substrate for BCRP (ABCG2) but not for P-gp (P-glycoprotein), suggesting the potential role of BCRP similar to that of MRP2 [71]. Furthermore, *in vitro* studies suggest that OTA alters the intestinal barrier and absorption functions [72].

### 4.2. Distribution

#### 4.2.1. Binding to Plasma Proteins

Albumin is the most abundant plasma protein in the circulatory system. Albumin binds OTA with unusually high affinity; therefore, 99.8% of OTA is in albumin-bound form in the human circulatory system [28]. Erythrocytes contain only traces of OTA [67]. It has been shown that the primary binding site of OTA on human serum albumin (HSA) is located on subdomain IIA (Sudlow’s Site I) [24,73,74,75]. There is a secondary binding site on subdomain IIIA (Sudlow’s Site II) but with much less affinity, suggesting its negligible relevance regarding the toxicokinetics of OTA [76]. The primary binding site of OTA is almost identical to that of warfarin (also presenting a coumarin backbone) [77]. The isocoumarin moiety of OTA is localized in an apolar cavity among amino acids A291, L238, I260, I264, I290, R257, and S287, while the phenyl group is surrounded by K199, H242, Y211, L238, and W214 amino acids [75]. The oxygen atoms of the carbonyl and phenolic hydroxyl groups orientate towards R257, while the carboxylic group towards R218 and/or R222 amino acids; data obtained for modified albumin strongly support the pivotal role of R257 and R218 arginines in the interaction [75]. The R257 arginine can deprotonate the phenolic hydroxyl group of OTA resulting in the formation of a very stable ion pair with HSA; this explains why the dianionic form of OTA is bound to HSA (even if the monoanionic form binds, it is rapidly deprotonated).

Apart from albumin, some other proteins of a molecular mass of about 20 kDa have been described which exert even higher binding affinity than albumin does [78,79]. These proteins are of much less concentration than albumin; however, they might have a potential role in the pathogenesis of BEN because of their free filtration through the glomeruli.

#### 4.2.2. Tissue Distribution

The tissue distribution of OTA is species-dependent and is also largely influenced by many factors including the amount of toxin, the way of ingestion, the composition of the diet, and the overall health status of the body. However, the major targets are the kidneys and the liver [1,3], skeletal muscle, fat tissue, and the brain also contain lesser amounts of the toxin, as well [80]. A possible explanation for the vulnerability of the kidneys and the liver might be explained by their special transport mechanisms. In the kidneys organic anion transporters (OATs), while in the liver organic anion-transporting polypeptides (OATPs), are the major molecular structures responsible for the active cellular uptake of OTA (OATs and OATPs are membrane transport proteins, belonging to the solute carrier transporter family) [81,82,83]. While basolateral OATs are mainly responsible for the uptake of OTA from blood into the tubule kidney cells, the apical OAT4 transporter may be involved in the urinary reabsorption of OTA resulting again its accumulation in tubule kidney cells [83]. Some of the kidney-specific OATPs can take part in the cellular uptake of OTA, as well [84]. Furthermore, low doses of OTA led to the increased expression of more organic anion transporter isotypes in rat kidneys [85]. In the proximal tubule cells a 62 kDa protein was identified with strong toxin-binding characteristics which might facilitate the tissue accumulation of the toxin [79].

Since under physiological circumstances OTA is present as a charged molecule, it crosses the placenta most probably by active transport mechanisms. Unfortunately, OTA levels are higher in the placenta and also two-fold higher in the fetus’s blood than those of the mother [86]. *In vitro* experiments suggest the possible involvement of OAT4 transporter [87].

### 4.3. Biotransformation

**Figure 2:**
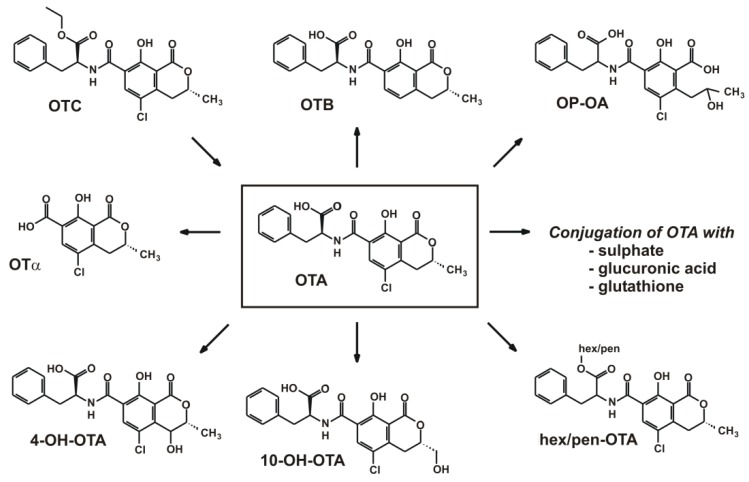
Biotransformation of Ochratoxin A.

Previous studies suggested that most of OTA remains unchanged, and that liver is not the sole organ to metabolize OTA [88]. Nevertheless, OTA can be biotransformed by both phase I and phase II enzymes. The majority of the metabolites (Figure 2) show low or no toxicity. In the gut, part of OTA is hydrolyzed to Ochratoxin α (OTα) by the action of proteolytic enzymes and by enzymes of the bacterial microflora [89,90,91,92]. Another possibility of the hydrolysis of OTA is opening the lactone ring under alkaline conditions that results in the formation of a highly toxic compound called lactone-opened OTA (OP-OA) [93]. 4-hyroxyochratoxin A (4-OH-OTA) is a product of microsomal oxidation of the toxin with low toxicity [94,95,96,97,98], while another less toxic product is 10-hydroxyochratoxin A (10-OH-OTA) [99,100,101,102]. Moreover, the formation of further hydroxyl metabolites of OTA was also observed in different species [103]. These phase I-type reactions are most probably related to the action of the CYP450 enzyme family [95,104,105,106,107,108,109]. Some studies suggest that after dechlorination OTA is transformed to Ochratoxin B with less genotoxicity [39,110,111,112,113]. Among phase II reactions sulfate, glucuronide, hexose/pentose (hex/pen-OTA), and glutathione (OT-GSH) conjugations of OTA are described as well [114,115,116,117,118]. The above listed metabolites have been observed in tissues, blood, and urine of animals and/or humans. Furthermore, *in vivo* studies suggest that there are sex differences regarding the carcinogenicity of OTA originating from the variation of OTA biotransformation in animals [15,33,37,119,120,121].

### 4.4. Excretion

#### 4.4.1. Renal Excretion

Due to the strong albumin binding of OTA, its elimination by glomerular filtration is negligible. Excretion of OTA is primarily done through tubular secretion. The tubular reabsorption of the toxin might be considered to be partially responsible for the intracellular accumulation of OTA [1]. Both *in vitro* and *in vivo* experiments suggest the importance of organic anion transporters [122,123,124]. Human OAT1 in the kidneys, while OAT3 transporter in the liver and brain, are considered as active members in the uptake of OTA from blood into tissues [81]. MRP2 transporters may play a role in the transport of OTA from kidney tubule cells into the urine [125,126]. Unfortunately, *in vivo* studies verify that the toxin is able to be reabsorbed from practically any part of the nephron both by active transport and by passive diffusion in a pH-dependent fashion [127].

#### 4.4.2. Fecal Excretion and Entero-Hepatic Circulation

Biliary excretion of OTA and its metabolites is the major route in its fecal excretion; however, secretion into the small intestine is also present. Intestinal mechanisms might involve MRP2 and BCRP transporters [70,71,128,129,130,131].

In animals, especially in rodents, the role of entero-hepatic circulation of OTA has been demonstrated [68,69,128,132]. In mice, the biliary excretion of the conjugated form of the toxin was observed; OTA possibly absorbs again after the hydrolysis of its conjugate by the bacterial microflora [69]. In addition to the strong albumin binding property, entero-hepatic circulation might enhance the slow elimination of OTA from the body.

#### 4.4.3. Excretion through Breast Milk

Although a great fluctuation is described for the toxin concentration in milk [133,134], there is a direct relationship between the ingestion of OTA and its concentration in the milk [135]. In a human study it was observed that the highest OTA level was found in breast milk during the first few days after delivery [136].

## 5. Mode of Action of OTA

### 5.1. Inhibition of Protein Synthesis

OTA is an inhibitor of protein synthesis in both *in vivo* and *in vitro* models. It was verified that OTA can inhibit the activity of phenylalanine t-RNA synthase [137,138]. First it was hypothesized that the phenylalanine moiety of OTA has a major role as a competitor between phenylalanine and the toxin. However, further studies showed that the isocoumarin structure is more important in this interaction than the phenylalanine moiety because modification of the isocoumarin structure has a significant impact on this action [139,140]. Docking studies have also supported the slight importance of the phenylalanine part [141]. Furthermore, OTA is also an inhibitor of phenylalanine hydroxylase; the toxin behaves as a false substrate of the enzyme because the hydroxylation of its phenylalanine moiety results in tyrosine-containing OTA which was detected even in *in vivo* experiments [142]. However, we have to note that the effects of OTA on phenylalanine t-RNA synthase and phenylalanine hydroxylase, *in vivo*, were observed after the treatment with relatively high OTA doses [137,142]. In addition to these non-specific ways of protein synthesis inhibition, OTA may influence the transcription of many proteins resulting in specific intracellular effects [143].

### 5.2. Inhibition of Cellular Energy Production

OTA has a strong negative effect on cellular energy (ATP) production [144]. Mitochondrial dysfunction is an early sign of toxicity [145] resulting in an overall decrease in protein synthesis. There are some key enzymes in gluconeogenesis e.g., phosphoenolpyruvate-carboxykinase (PEPCK) which shows decreased activity due to OTA exposure [146,147]. It was also proven that OTA interferes with the expression of PEPCK at the mRNA level [148,149]. Furthermore, the toxin can penetrate into the mitochondria and most probably binds to proteins involved in maintenance of the membrane potential and the oxidative phosphorylation by interfering with phosphate transport and by inhibition of electron transport as well [150,151,152].

### 5.3. Genotoxic Effect

**Figure 3:**
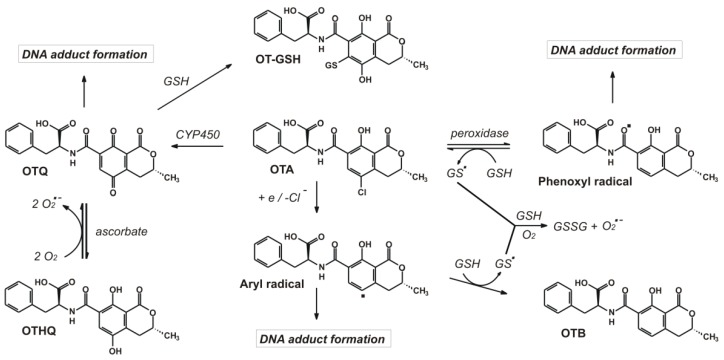
Bioactivation of Ochratoxin A producing genotoxic metabolites based on Pfohl-Leszkowicz and Manderville [38] (OTA: Ochratoxin A, OT-GSH: OTA-glutathione conjugate, OTQ: OTA-quinone, OTHQ: OTA-hydroquinone, and OTB: Ochratoxin B).

Several experiments suggest that OTA has genotoxic effects [38]. Following bioactivation, electrophilic products are formed from the toxin which can covalently bind to DNA causing mutations and subsequent formation of malignant tumors. Figure 3 summarizes the mechanisms of adduct formations detailed below.

A common principle for the different theories based on the parachlorophenol (PCP) structure found in OTA [153] is that PCP can undergo CYP450-catalized oxidative dechlorination, resulting in a quinoidal structure that can bind covalently to thiol groups as well as 2′-deoxyguanosine (dG), or other adducts may form [154,155,156]. Oxidation of OTA by CYP450 enzymes also produces a reactive electrophilic product called OTA-quinone (OTQ). OTQ can be partially detoxified by conjugation with GSH or it possibly forms OTA-hydroquinone (OTHQ) after reduction [117,157,158]. OTHQ was detected both in rat and in human urine samples [15,37,121,159].

Another theory considers the activation by peroxidases as an important step to produce the phenoxyl radical from OTA [160,161]. Then, in the presence of glutathione, the phenoxyl radical may be converted to OTA again, but at the expense of the formation of a superoxide anion radical (O_2_^•^¯) [162,163]. O_2_^•^¯ forms H_2_O_2_ which can induce a Fenton reaction resulting in the appearance of a hydroxyl radical (OH^•^), causing oxidative damage again. This mechanism might partly explain the observation that OTA depletes GSH in cellular models [164]. Furthermore, the phenoxyl radical may directly form C8-deoxyguanosine-adducts (C8-dG) [165,166].

Reductive dechlorination of OTA may produce reactive aryl radicals [167] resulting in the formation of C8 purine nucleotide adducts [168,169,170].

**Figure 4:**
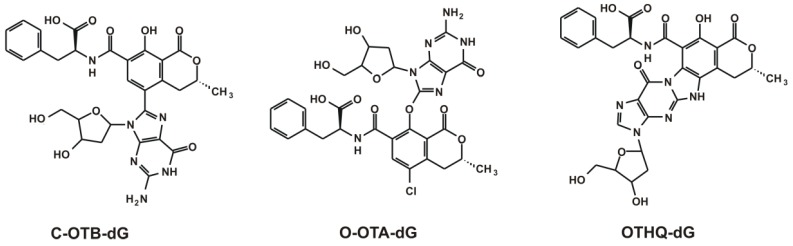
Chemical structures of OTA nucleoside adducts based on Pfohl-Leszkowicz and Manderville [38] (C-OTB-dG: C-coupled C8-deoxyguanosine-adduct, O-OTA-dG: O-coupled C8-deoxyguanosine-adduct, and OTHQ-dG: OTA-hydroquinone C8-deoxyguanosine-adduct).

Figure 4 demonstrates the chemical structure of the potentially-occurring dG adducts. Photoreaction of OTA in the presence of dG and further reactions in the presence of OTA, dG, Fe^2+^, or horseradish peroxidase/H_2_O_2_ resulted in C-coupled C8-dG-adducts (C-OTB-dG) [171]. C-OTB-dG adduct may be produced through phenoxyl and aryl radical pathways; however, the phenoxyl radical of OTA may also lead to the formation of O-coupled C8-dG-adducts (O-OTA-dG) [172,173]. Finally, the third potential compound is the OTHQ-dG-adduct [37,117]. In spite of much *in vitro* and *in vivo* experimental data obtained by isotope and LC-MS techniques, there are controversies about the presence of DNA adducts [174,175]. C-OTB-dG adduct formation might occur with the highest probability [176] but several authors seriously query the presence of DNA adducts due to OTA exposure [177,178,179]. A recent study suggests that OTA alone does not lead to DNA adduct formation in rats; however, the co-treatment of aristolochic acid with OTA results in higher adduct formation than the treatment with aristolochic acid alone [180].

### 5.4. Induction of Oxidative/Nitrosative Stress

Several studies suggest that both *in vitro* and *in vivo* OTA exposure results in the overproduction of free radicals. Increased ROS production, as well as oxidative damage (lipids, proteins, and DNA) are described by many authors [1,181]. The formation of each OTQ or phenoxyl and aryl radicals can lead to increased ROS production as discussed previously (Figure 3). Furthermore, OTA can possibly induce lipid peroxidation using Fe^3+^ as cofactor. In the presence of NADPH-CYP450 reductase OTA-Fe^3+^ complex facilitates the reduction of Fe^3+^ and the formed OTA-Fe^2+^ complex initiates the appearance of free radicals leading to lipid peroxidation and DNA damage [182,183].

Recent data indicate that intracellular Zn^2+^ levels might also be in connection with OTA toxicity. OTA can deplete Zn^2+^ most probably by its action on transporter proteins and metallothioneins [184]. It was shown in liver cell cultures that OTA significantly increases ROS concentration and expression of several metallothioneins, while reducing superoxide dismutase (SOD) activity and catalase mRNA levels [185]. Furthermore, OTA is able to directly interact with Zn^2+^; the complex formation can also be responsible for the decreased presence of free intracellular zinc levels [186].

Moreover, OTA treatment causes not only increased ROS production but also reduces the antioxidant defense of cells by lowering activator protein 1 (AP-1) and nuclear factor-erythroid 2 p45-related factor 2 (Nrf2) activation, which molecules regulate the transcription of glutathione, glutathione *S*-transferase (GST), and further cytoprotective enzymes [187,188,189]. Recent studies highlighted that OTA can also inhibit the expression of Nrf2 protein, its translocation into the cell nucleus, as well as its binding to DNA [190].

It is important to note that the reactive nitrogen species levels may also increase in OTA-treated cells. OTA facilitates the expression of inducible nitrogen oxide synthase (iNOS) enzyme and also increases the expression and activity of dimethylarginine dimethylaminohydrolase (DDAH) with subsequent elevation of nitrogen monoxide (NO) synthesis and increased nitrite/nitrate concentrations [181,191]. High levels of NO may cause nitrosative stress because it can react with O_2_^•^¯ resulting in the formation of peroxynitrite (ONOO¯), which in turn gives nitrogen dioxide (NO_2_) and hydroxyl radicals (OH^•^).

Nevertheless, some studies suggest that the carcinogenic effect of OTA is independent from oxidative damage. In these studies, OTA-induced increased cell proliferation, cell cycle aberration, and apoptosis were observed in rats [192,193].

### 5.5. Apoptosis

OTA can cause both apoptotic and necrotic cell death [1,181]. Even at nanomolar concentration apoptosis markers could be observed, e.g., DNA fragmentation, chromatin condensation and increased caspase-3 activity [194]. Altered expression of different genes could be a potential cause of apoptosis: marked transcriptional changes of many genes were observed which are involved in DNA damage response and apoptosis (e.g., GADD153, GADD45, clusterin, and p53) [192,195]. The important protective role of p53 regarding the carcinogenic effect of OTA-treated mice was demonstrated by Kuroda *et al.* [196]. Regulation of signal transduction pathways can also be a possible reason of apoptosis induction; activation of MAPK-, ERK-, p38, and JNK were described in kidney and/or liver cells [194,197,198]. Interestingly, ERK is activated only weakly, and p38 is not activated at all, by OTA in human kidney cells; despite OTA being a strong activator of MAPK, ERK, and p38 in rat liver cells [197]. In a recent study, the sustained activation of c-MET/PI3K/Akt and MEK/ERK1-2 signaling pathways were reported in human kidney cells [199]. Another study suggests the role of apoptosis signal-regulating kinase 1 (ASK1) which can activate JNK and p38 pathways, as well as it has a pivotal role in oxidative stress- and in endoplasmic reticulum stress-induced cell death [200]. The probability of the type of OTA-induced cell injury (apoptosis or necrosis) most likely depends on the applied concentration of the toxin [198]. Oxidative stress may also play a role in cell death [201,202]; however, its relevance is still unclear. In other experiments, increased pro-inflammatory mediator levels (TNFα- and IL-6) were observed in rat liver perfusates of OTA-treated rats [203,204], where the NF-κB pathway might also be involved [205].

### 5.6. Influence on Mitosis

Several researchers consider OTA to be a non-mutagenic agent with no direct effect on DNA. They explain the OTA-induced carcinogenesis by causing disruptions of mitosis and chromosomal instability [179,206,207,208,209]. In human kidney cells, the block of the metaphase/anaphase transition, aberrant mitotic formations, and giant cells were observed [206,208]. Disorganization of the microtubular system and inhibition of histone acetyl transferase (HAT) enzymes were also detected [209]. Since HATs are responsible for the regulation of gene expression, DNA repair, and cell cycle control, their inhibition can lead to disruption of mitosis, cell proliferation and genetic instability [179].

### 5.7. Induction of Cell Cycle Arrest

Several studies have shown the negative effects of OTA on cell cycle in kidney cells and in lung fibroblasts [164,210,211]. Selective G2 phase arrest was observed in OTA-treated gastric epithelial cells [45]. Negative effects of OTA on cyclin-CDK (cyclin-dependent kinase) system were established: decreased expression of CDK25, CDK2, and cyclin B1 were observed both at the protein and at the mRNA levels, and the amount of the cyclin B1-CDK2 complex was reduced as well. It appeared that activation of ERK and p38 plays an important role in the above mechanism [212]. The role of free radicals cannot be underestimated because *N*-acetyl cysteine (NAC) supplementation effectively abolished OTA-induced cell cycle arrest [213]; the authors suggest the involvement of ATM-Chk2 and ATM-p53-p21 signaling pathways. Downregulation of Cdc2 and cyclin B1, as well as consequential G2 phase arrest, were also observed in esophageal cells [46]. In another study performed with human peripheral blood mononuclear cells, OTA caused G1 phase cell cycle arrest due to the decreased CDK4 and cyclin D1 expression; NAC alleviated again this unpleasant effect [214]. Furthermore, decreases of both mRNA and protein levels of cyclin A2, cyclin E1, and CDK2 were observed in human embryonic kidney cells after treatment with OTA, resulting in S-phase cell cycle arrest [215]. These negative consequences were alleviated with the pretreatment of cells with NAC [216].

### 5.8. Other Mechanisms

Few previous studies suggest that, due to lipid peroxidation in OTA-treated cells, the permeability towards Ca^2+^ increases and also the intracellular calcium stores may be depleted [217,218]. Hoehler *et al.* [219,220] found in kidney cells that increased intracellular Ca^2+^ in the presence of OTA causes uncoupling of oxidative phosphorylation and decreases ATP synthesis. OTA, even in nanomolar concentrations, negatively affects Ca^2+^- and cAMP homeostasis interfering with calcium signaling which, in turn, results in abnormal cell proliferation [221]. Another possible explanation of the increase in total intracellular calcium is the characteristic shrinkage of toxin-treated cells. It has been postulated by Dopp *et al.* [218] that OTA binds irreversibly to actin filaments causing their shortening and aggregation.

OTA is able to directly interact with different alkali and alkaline earth metal ions; low stability complexes are formed with K^+^, Na^+^, and Li^+^ ions while the presence of more stable complexes of OTA were observed with Ba^2+^, Ca^2+^, and Mg^2+^, in this order [26]. Each ion showed higher preference towards the dianionic form of OTA. Similarly with OTA-Zn^2+^ interaction [186], the biological importance of these complex formations are yet unclear.

## 6. Protective Agents—Overview

There have been some attempts to inhibit the absorption of OTA from the gastrointestinal tract by using adsorbents [19,222,223]. However, these methods are not selective and cause problems in long-term usage. The anion exchange resin cholestyramine can bind OTA in the gut, therefore inhibiting its absorption, enhancing its fecal elimination, and disrupting the enterohepatic circulation of OTA [224,225]. Application of NaHCO_3_ favors ionization of the toxin, decreasing its gastrointestinal absorption and increasing its elimination through the urine [226]. However, long-term usage of both cholestyramine and NaHCO_3_ is not recommended. Piroxicam is a substrate of OAT and may compete with OTA for its uptake into kidney cells [227]. However, larger doses of piroxicam also lead to nephrotoxic consequences. Application of the enzyme inductor phenobarbital resulted in controversial outcomes in different studies [102,228,229,230]. Phenylalanine and aspartame may counteract with the nonspecific effects of OTA in reducing protein synthesis due to the competitive action of the phenylalanine moiety [137,231,232,233]. However, contradictory results regarding phenylalanine exist as well [234].

There have been many trials to reduce the effects of OTA by administration of antioxidants prior, simultaneously, and after toxin exposure in cellular and in animal models. Application of SOD and catalase enzymes or the support of the glutathione system by NAC attenuated some negative effects of OTA (e.g., cytotoxicity, cell cycle arrest, or DNA damage) in several cases [213,214,216,235]. In other studies NAC only poorly alleviated the OTA-induced toxicity [113,236]. In cellular models vitamin C, α-tocopherol (vitamin E), and retinol (vitamin A) supplementation proved to be successful as well [237,238,239,240]. Pretreatment or co-treatment of OTA-exposed cells with different polyphenolic and other natural compounds showed positive effects on cell viability [181]. Pretreatment of kidney cells with epigallocatechin gallate (EGCG) increased the proliferation rate and decreased ROS levels and DNA fragmentation [241]; however, simultaneous exposure with the toxin or exposure after OTA treatment did not cause attenuation of toxicity. Co-exposure with rosmarinic acid of liver cells resulted in decreased ROS production and improvement in viability with less inhibition of protein and DNA synthesis [242]. Similar beneficial effects were found during the pretreatment or co-treatment of gingival fibroblasts, colon epithelial and liver cells with cyanidin 3-*O*-beta-d-glucoside (C3G) [243,244]. Animal experiments showed that C3G could attenuate OTA toxicity by influencing increased DDAH- and iNOS activation in rats, and in this way counterbalancing nitrosative stress [245]. Silibinin effectively decreased OTA-induced apoptosis in hepatocytes [246]. Luteolin, chlorogenic acid, and caffeic acid attenuated the OTA-mediated viability loss in kidney cells and lymphocytes while decreasing OTA-induced DNA damage of the blood cells of BALB/c mice [247]. Quercetin pretreatment suppressed OTA-induced cytotoxicity and oxidative stress and prevented OTA-induced apoptosis by lowering the activation of caspase cascade, DNA fragmentation, and micronucleus formation in kidney and in liver cells; these positive effects were attributed to the activation of Nrf2 pathway by quercetin causing the protection against OTA-induced alteration of the antioxidant defense [248,249]. Furthermore, diosmetin completely abolished the OTA-induced ATP depletion in kidney cells [144]. Carotenoid lycopene alleviated OTA-induced DNA damage, oxidative stress and apoptosis in rats [250,251]. Treatment with certain plant extracts also resulted in beneficial impacts in animal experiments [181,252,253]. Since OTA depletes intracellular zinc levels, zinc supplementation might be beneficial [185]. Furthermore, selenomethionine alleviated OTA-induced toxicity in kidney cells, the positive impact is possibly caused by the improvement of the expression of some selenoenzymes [254].

Despite of the presence of several data from *in vitro* and from animal studies, we have no strict evidence regarding the protective effect of the above-listed agents. Many hypotheses exist on the main toxic mechanism of OTA; however, even nowadays our knowledge is limited. Since we do not know the exact target of OTA in human kidney cells responsible for the toxic consequences after the long-term exposition with the mycotoxin, both the prevention and the treatment of OTA-induced toxicity are very difficult. Furthermore, the application of these protective agents can be problematic, in general, because of poor gastrointestinal absorption, high presystemic elimination, or insufficient tissue distribution. On the other hand, enhancement of the elimination of OTA from the body may be a promising tool to reduce the long-term exposition of target cells.

## 7. Displacement of OTA from Albumin

In our previous experiments three different models were applied for the calculation of the association constant (*K*) of OTA-HSA complex which showed log*K* values at about 7.3–7.6 [25,31,32]. However, OTA-albumin binding of different species is not uniform. Human serum albumin (HSA) seems to exert a very strong binding while, e.g., bovine (BSA) or rat serum albumin (RSA), show less affinity for the toxin [24,31]. In our previous study, the association constants of OTA-HSA, OTA-BSA, and OTA-RSA were determined (log*K*_OTA-HSA_ = 7.65 ± 0.36, log*K*_OTA-BSA_ = 6.48 ± 0.22 and log*K*_OTA-RSA_ = 6.17 ± 0.12) [31]. Thus, the stability of OTA-HSA complex is 15-fold and 30-fold higher compared to those of OTA-BSA and OTA-RSA complexes, respectively. This observation perfectly explains why we notice, by far, the longest elimination half-life of OTA in humans [28]. A previous study with albumin-deficient rats showed the very high importance of albumin binding regarding the slow elimination of OTA [29]. Furthermore, a recent study with rats also proved that the accumulation of OTA in the kidney is due to strong binding of OTA to plasma proteins and its long half-life in plasma [255]. It is very likely that, in humans, this impact could be more dominant because of the substantially stronger interaction of OTA with human albumin.

If we displace a considerable amount of OTA from albumin, its increased elimination rate might reduce the chronic exposure of the target cells (mainly kidney tubule and liver cells). In this case, the free (not albumin-bound) OTA concentration will increase both in blood and in urine. Since the total concentration of OTA in the blood is not so high, it is very unlikely that the displacement will cause acute toxicity. OTA is transported to kidney and liver cells through active transport proteins. Due to the half-maximal transport rate of OTA regarding organic anion transporters is relatively low [81], the cellular uptake of OTA may become capacity-limited in the presence of other endogenous and exogenous substrates of these transporters. On the other hand, the half-maximal transport rate of OTA through OATP is high (approximately 20 µM) [82]; therefore, it is very unlikely that the uptake of OTA by OATP will become capacity-limited. Protective effects of nonsteroidal anti-inflammatory drugs piroxicam and indomethacin against the toxic impacts of OTA could be partly caused by the competitive displacement of OTA from albumin [227,256,257]. Co-treatment with phenylbutazone caused negative result in some studies [258]; however, our previous investigation revealed that phenylbutazone is a poorly effective competitor in this context [30]. In addition to piroxicam, aspartam is also able to effectively displace OTA from HSA [259]. After six-week pretreatment of rats with OTA, administration of aspartame for 10 days resulted in the strong decrease of OTA in the blood and kidneys [235]. Furthermore, lower levels of OTA in the brain, liver, and testicles were observed, as well as relatively higher rates of poorly-toxic metabolites of OTA (Ochratoxin α and hydroxylated OTA derivatives) were found. These results also strongly suggest the protective effect of displacement of OTA from albumin. In our previous study, we demonstrated that besides warfarin and the above-listed drugs glipizide, simvastatin, and mainly furosemide, are also able to compete with OTA for binding to HSA [30]. In our opinion, it would be highly interesting to examine the occurrence of BEN and urinary tract tumors in patients who have been continuously treated with these drugs and live in the endemic areas.

Application of naturally-occurring flavonoids for the same purpose could also be very promising. As we demonstrated, flavonoid aglycones are able to displace OTA from HSA very effectively; they are much stronger competitors than the drug molecules tested [25,30]. Most of the flavonoids are therapeutically safe and, besides the displacement of OTA from albumin, they may have further positive effects (see in Section 6). Furthermore, some flavonoids are able to inhibit different OAT and OATP transporters [260,261,262] which take part in the cellular uptake of OTA. In contrast, we also need to mention that certain flavonoids can inhibit the MRP2 transporter as well, which can decrease the excretion of OTA [70,263,264]. The first-pass metabolism of flavonoids is high, and it results in their limited pharmacological effects [265]. On the other hand, previous studies highlighted that many flavonoid metabolites also show (even similar or higher) albumin binding properties compared to the parent compound [266].

## 8. Conclusions

As we discussed above, the mechanism of action of OTA is still unclear; therefore, it is difficult to develop effective strategies in order to alleviate the OTA-induced toxicity at the cellular level. On the other hand, disruption of the normal toxicokinetics of OTA, e.g., inhibition of its cellular uptake or enhancement of its elimination from the body, could be promising techniques to prevent the cellular accumulation of OTA as well as its toxic action. Obviously, animal experiments are needed to decide if displacement of OTA from albumin and its increased elimination have a positive impact on attenuating OTA toxicity. However, it should be kept in mind that binding of OTA to albumin is strongly species-dependent; therefore, to find a proper animal model is essential. Based on these considerations, we suggest that further investigations of agents which are able to positively influence the toxicokinetics of OTA is a very important issue, and it might provide a more suitable way to prevent and/or treat the OTA-induced unpleasant consequences, especially in the endemic areas of increased OTA exposure.

## Abbreviations

ATP: Adenosine triphosphate
BCRP: Breast Cancer Resistance Protein
BEN: Balkan Endemic Nephropathy
C8-dG: C8-deoxyguanosine DNA-adduct
CYP450: Cytochrom P450 enzyme family
DDAH: Dimethylarginine dimethylaminohydrolase
GSH: Glutathione (reduced)
GSSG: Glutathione (oxidized)
HSA: Human serum albumin
iNOS: Inducible Nitrogen Oxide Synthase
MRP2: Multidrug Resistance-associated Protein 2
NAC: *N*-acetyl cysteine
Nrf2: Nuclear factor-erythroid 2 p45-related factor 2
OAT: Organic Anion Transporter
OATP: Organic Anion-transporting Polypeptide
OTα: Ochratoxin α
OTA: Ochratoxin A
OTB: Ochratoxin B
OTC: Ochratoxin C
OTHQ: OTA-hydroquinone
OTQ: OTA-quinone
PEPCK: Phosphoenolpyruvate carboxykinase
ROS: Reactive oxygen species
SOD: Superoxide dismutase
